# Subtle morpho-functional kidney changes in type-2 diabetes mellitus patients: A duplex ultrasound assessment

**DOI:** 10.12669/pjms.38.3.4699

**Published:** 2022

**Authors:** Amal A. Alareqi, Sultan Abdulwadoud Alshoabi, Abdulaziz A. Qurashi, Abdullgabbar M. Hamid

**Affiliations:** 1 Amal A. Alareqi, Department of Radiology, University of Science and Technology Hospital (USTH), Sana’a, Republic of Yemen. Radiology Department, 21 September University of Medical and Applied Sciences, Sana’a, Republic of Yemen; 2 Sultan Abdulwadoud Alshoabi, Department of Diagnostic Radiology Technology, College of Applied Medical Sciences, Taibah University, Almadinah Almunawwarah, Kingdom of Saudi Arabia; 3 Abdulaziz A. Qurashi, Department of Diagnostic Radiology Technology, College of Applied Medical Sciences, Taibah University, Almadinah Almunawwarah, Kingdom of Saudi Arabia; 4 Abdullgabbar M. Hamid Department of Radiology, Rush University Medical Center, Chicago, IL, United States America

**Keywords:** Subtle kidney changes, Doppler dynamic measurements, Renal length, Renal resistive index, Pulsatility index

## Abstract

**Objectives:**

Diabetes mellitus (DM) is a common endocrine disease with serious effects on multiple organs including the kidneys. This study aimed to investigate the subtle effects of type 2 DM (T2DM) on the kidneys.

**Methods:**

This was a prospective case-control study conducted in the Radiology Department of University of Science and Technology Hospital (USTH) campus, Sana’a, Republic of Yemen, from 1 January 2020 to 31 November 2020. The renal length (RL), renal width (RW), resistive index (RI), and pulsatility index (PI) were prospectively measured in patients with T2DM and healthy controls. The results were compared using the independent samples t-test. Comparisons were likewise performed between patients with controlled DM and patients with uncontrolled DM.

**Results:**

A total of hundred individuals, 50 diabetic patients and 50 controls, were enrolled in this study. Their mean age was 54 ± 7.88 years (range: 40–75 years). The RL, RI, and PI of both kidneys were significantly higher in T2DM than in the control group. Moreover, the RL, RI, PI and creatinine were slightly higher in patients with uncontrolled than in those with controlled DM.

**Conclusion:**

T2DM has significant accentuating effects on the RL, RI and PI associated with low effective renal plasma flow, even before acute kidney injury or chronic kidney disease diagnosis, which may be attenuated by careful regulation of DM. Ultrasound Doppler is a highly valuable imaging modality for evaluating the subtle effects of T2DM on kidney dimensions and blood flow. The RI can be implemented as a tool for the early diagnosis of kidney disease and contribute to slowing the disease progression and preventing renal failure.

## INTRODUCTION

Diabetes mellitus (DM) is a common disease with serious effects on multiple organs, including the kidneys. DM leads to micro- and macrovascular kidney disease and increased susceptibility to infection. It predisposes patients to renal infection, renal or perirenal abscess, or emphysematous pyelonephritis. In long term, it can lead to diabetic nephropathy (NP), renal papillary necrosis, or renal artery stenosis (RAS).^1^

Ultrasound (US) plays a critical role in evaluation of chronic kidney disease (CKD), and is the imaging modality of choice for determining the cause of acute kidney injury (AKI) and understanding the renal pathophysiological characteristics.^2^,^3^

It is a non-invasive, inexpensive, widely available, and radiation-free imaging modality that can determine the morphology of the kidney by measuring the renal length (RL), renal width (RW), renal volume and parenchymal thickness. It can also determine kidney function by measuring the RL, RW, parenchymal thickness, and parenchymal echogenicity.^4^ Bipolar RL is a reliable measure of kidney size in both healthy adults and CKD patients.^5^,^6^

Duplex US can help determine renal function by identifying large renal arterial and venous abnormalities and the renal resistive index (RI), and pulsatility index (PI). The renal RI is a recently suggested method for assessing renal perfusion, detecting RAS, evaluating the risk of progression in CKD, and predicting the renal outcome in AKI. In diabetic CKD, a higher renal RI correlates with vascular and interstitial kidney damage and is a reliable predictor of disease progression.^7^

The RI and PI are calculated from the blood flow velocity in renal arteries during the cardiac cycle and used as measurements of downstream resistance in the arteries to detect peripheral vascular diseases. They increase with higher renal vascular resistance and they are useful in the investigation and monitoring of RAS. High RI and PI are associated with low effective renal plasma flow.^8^

Because of lack of previous studies about the subtle changes of the kidneys of DM patients before clearly diagnosing diabetic NP, this study was designed to evaluate early subtle changes of the kidneys of DM patients with no obvious kidney disease, by comparing their renal measurements to those of non-diabetic individuals.

## METHODS

This was a prospective case-control study involving DM patients and non-DM controls conducted in the Ultrasound unit of the Radiology Department of University of Science and Technology Hospital (USTH) campus, Sana’a, Republic of Yemen, from 1 January 2020 to 31 November 2020.

Type-2 diabetes mellitus (T2DM) patients referring for US imaging of the kidneys who were more than 40 years old and provided informed consent were enrolled in this study. Patients with known CKD or AKI, patients on haemodialysis, kidney transplant patients, and solitary kidney patients were excluded.

Ultrasound (US) and Doppler were performed with the same Ultrasound machine (Siemens Acuson X600 Ultrasound System) using the standard gray-scale B-mode with a curved array transducer of 2–5 MHz and a standard protocol. The gain and time gain compensation of US images were adjusted manually. All participants were examined by the same radiologist with five years’ post doctorate experience in US Doppler imaging.

**Fig.1:**
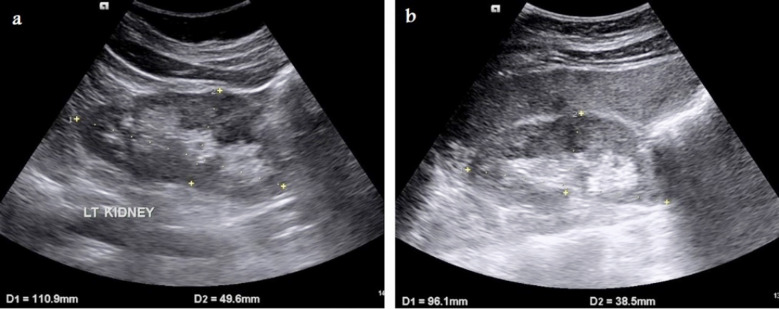
Selected gray-scale ultrasonography longitudinal sections of both kidneys in a 45-year-old female diabetic patient referred for follow up ultrasound imaging that show normal renal measurements (length and width) of a) left kidney, and b) right kidney.

**Fig.2:**
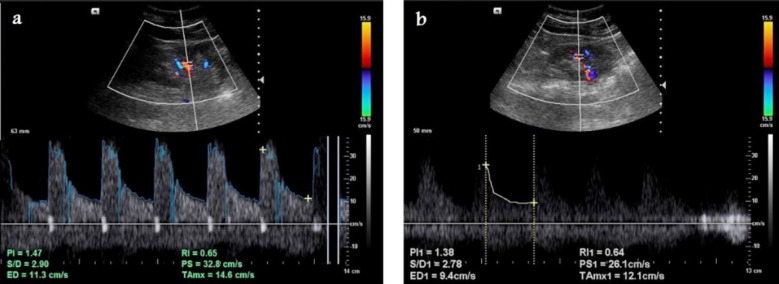
Selected spectral Doppler ultrasound images of the same 50-year-old female diabetic patient referred for follow up ultrasound imaging show Doppler measurement of RI (a) automatically, and (b) manually that was slightly higher than normal.

The length of the right and left kidneys was measured in the visually estimated largest longitudinal section of each kidney. The width of both kidneys was measured in sections perpendicular to the longitudinal axis (Fig.1). The renal RI was measured either automatically or manually (Fig.2) using pulsed Doppler Ultrasound in the arcuate arteries at the level of the cortico-medullary junction or in the interlobar arteries according to the following equation^9^:


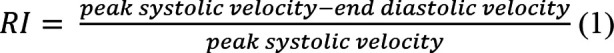


The renal PI was measured according to the following equation^9^:


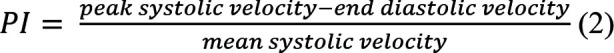


The parenchymal echogenicity of both kidneys was assessed by applying low tissue harmonic. Creatinine was investigated only in DM patients. The body mass index (BMI) of the participants was measured according to the following equation^10^:


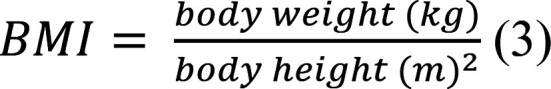


### Statistical Analysis

Data analysis was performed using IBM SPSS Statistics version 25, (IBM Corp., Armonk, NY, USA). Means and standard deviations were calculated for age, BMI, RL, RW, RI, PI, creatinine and duration of DM. The renal measurements in the DM patient group were compared to those in the non-DM (control) group using the independent samples t-test. Comparisons were likewise performed between patients with controlled DM and patients with uncontrolled DM according to the last measurements of haemoglobin A1c (HbA1c). A p-value of less than 0.05 was considered statistically significant.

### Ethical Approval

This study was approved by the Research Ethics Committee of USTH under a protocol issued on 26-4-2019. Informed consent was obtained from all participants. Anonymity was safeguarded during and after the study.

## RESULTS

A total of one hundred individuals, 50 DM patients and 50 controls, were enrolled in the study. Their mean age was 54 ± 7.88 years (range: 40–75 years). Females accounted for 74% and males accounted for 26% of the participants. The mean BMI was 29.51 ± 7.32 in the patient group and 25.65 ± 6.05 in the control group.

**Fig.3:**
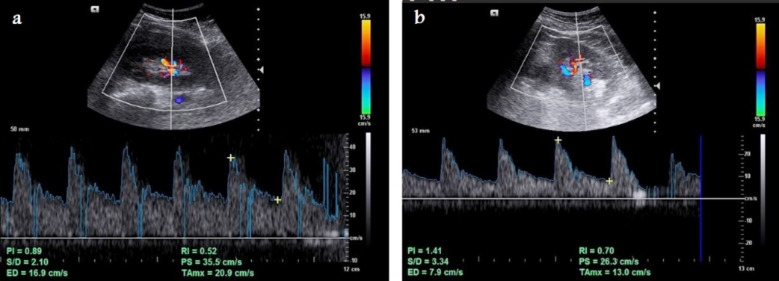
Selected spectral Doppler ultrasound images show Doppler RI of a) normal 52-year-old female individual with normal low resistance waveforms with RI=0.52, and b) diabetic 55-year-old female patient with RI=0.70 that is higher than normal.

**Fig.4:**
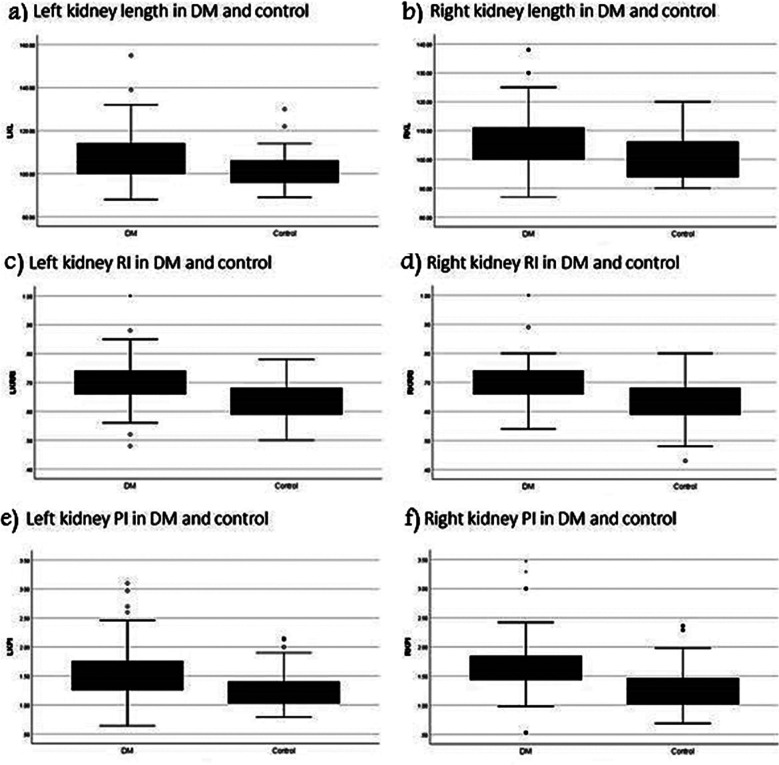
Boxplot shows significant increase of both a) left and b) right kidney length of DM patients than that of control group. Boxplot shows significant increase of renal re sistive index (RI) of both c) left and d) right kidney of DM patients than that of control group. Boxplot shows significant increase of pulsatility index (PI) of both e) left and f) right kidney of DM patients than that of control group.

The RL, RI, and PI of both the right and left kidneys were significantly higher in the DM than in the control group (Table-I, Fig.3 and 4). Moreover, the RI and PI of both the right and left kidneys were significantly higher, and creatinine was also higher in patients with uncontrolled DM than in those with controlled DM. There was no significant difference in BMI between patients with controlled DM and those with uncontrolled DM. The duration of DM had no effect on DM control status (Table-II).

**Table I:** Right and left kidney measurements in DM patients group and in control group.

Variables	Categories	Mean	Standard deviation	P-value	95% Confidence Interval
RKL	DM	106.0400	10.43143	0.011	1.10610-8.41390
	Control	101.2800	7.79073		
LKL	DM	108.0000	12.45891	0.003	2.32594-10.59406
	Control	101.5400	7.85899		
RKW	DM	44.9200	6.50852	0.092	-.40193-5.24193
	Control	42.5000	7.66452		
LKW	DM	48.3600	7.58559	0.184	-.90923-4.66923
	Control	46.4800	6.42139		
RKRRI	DM	.7074	.07575	<0.001	.04364-.10396
	Control	.6336	.07620		
LKRRI	DM	.6986	.08480	<0.001	.03690-.09670
	Control	.6318	.06448		
RKPI	DM	1.6912	.51654	<0.001	.20080-.55840
	Control	1.3116	.37291		
LKPI	DM	1.5646	.53666	0.001	.13710-.49332
	Control	1.2494	.32978		

Table shows significant differences in RL, RI, and PI between DM patients and normal individuals. DM: diabetes mellitus, RKL: right kidney length, LKL: left kidney length, RKW: right kidney width, LKW: left kidney width, RKRRI: right kidney renal resistive index, LKRRI: left kidney renal resistive index, RKPI: right kidney pulsatility index, LKPI: left kidney pulsatility index.

**Table II:** Right and left kidney measurements in patients with controlled DM and those with uncontrolled DM.

Variables	Categories	Mean	Standard deviation	P-value	95% confidence interval
RKL	Controlled DM	104.1429	5.27347	.239	-8.87429-2.37212
	Uncontrolled	107.3939	10.18866
LKL	Controlled DM	104.1429	8.35521	.186	-13.83499-2.96919
	Uncontrolled	109.5758	13.10541
RKW	Controlled DM	42.4286	8.69592	.292	-11.99280-4.18328
	Uncontrolled	46.3333	5.70453
LKW	Controlled DM	49.2857	7.38725	.974	-6.88663-7.09442
	Uncontrolled	49.1818	7.46431
RKRRI	Controlled DM	.6700	.06351	.093	-.11319-.01016
	Uncontrolled	.7215	.08228
LKRRI	Controlled DM	.6571	.06726	.143	-.11407-.01865
	Uncontrolled	.7048	.09605
RKPI	Controlled DM	1.3571	.39076	.026	-.83800--.06347-
	Uncontrolled	1.8079	.57161
LKPI	Controlled DM	1.2243	.38004	.024	-.83313--.06860-
	Uncontrolled	1.6752	.59678
Duration	Controlled DM	6.1429	8.45436	.412	-10.85833-4.95050
	Uncontrolled	9.0968	6.55416
Creatinine	Controlled DM	.7950	.24023	.117	-1.01294-.11894
	Uncontrolled	1.2420	1.29276
BMI	Controlled DM	30.2886	5.07164	.984	-5.15133-5.24981
	Uncontrolled	30.2393	8.19290

Table shows significant differences in RI and PI in patients with controlled and patients with uncontrolled DM. DM: diabetes mellitus, RKL: right kidney length, LKL: left kidney length, RKW: right kidney width, LKW: left kidney width, RKRRI: right kidney renal resistive index, LKRRI: left kidney renal resistive index, RKPI: right kidney pulsatile index, LKPI: left kidney pulsatile index, BMI: body mass index.

## DISCUSSION

DM is a chronic metabolic disease associated with serious complications in various organs. Metabolic changes in DM may lead to glomerular hypertrophy and sclerosis, tubulointerstitial inflammation and fibrosis, and NP onset and progression with many structural and functional changes in the kidneys.^11^ This study was conducted to determine early subtle morphological and haemodynamic changes in the kidneys of T2DM patients with no obvious acute or chronic renal disease by comparing their renal measurements with those of non-diabetic individuals. The comparisons revealed significant differences in RL, RI, and PI in both kidneys.

In this study, the BMI was significantly higher in the DM than in the control group. This finding is consistent with Friedman et al., who reported a strong relationship between T2DM and obesity, which is a risk factor for kidney disease and a leading cause of CKD.^12^ The relationship was explained by D’Agati et al., who found that the incidence of obesity-related NP is increasing with the increasing obesity rates worldwide. Obesity increases the glomerular filtration rate (GFR), renal blood flow, filtration fraction, and sodium reabsorption in renal tubules, leading to glomerulus enlargement and, consequently, kidney enlargement.^13^ Moreover, Min et al. reported a close relationship between obesity and T2DM, which is associated with a higher incidence of CKD. Weight reduction improves DM and renal outcomes and reduces the risk of kidney disease.^14^

In this study, we found a significantly greater RL in the T2DM than in the control group. This result is consistent with Umanath and Lewis, who found that kidney size and weight increase by an average 15% in DM patients and remain increased even after a decrease in kidney function.^15^ It is also consistent with Zerbini et al., who reported that renal hypertrophy and hyperfiltration become manifest soon after the onset of type 1 DM (T1DM), and persistent renal hypertrophy and a reduction in GFR precede microalbuminuria of diabetic NP in T1DM.^16^ Renal hypertrophy in diabetic patients has been attributed to thickening of the glomerular basement membrane and diabetic macroangiopathy with arterial hyalinosis in the afferent and efferent arterioles, which leads to glomerular hyperfiltration.^17^

We also found that the renal RI was significantly higher in the DM than in the control group. This result is in line with Afsar and Elsurer, who found that the RI is increased in most studies on T2DM.^18^ The RI is a valuable diagnostic tool for DM patients developing diabetic NP and strongly correlates with serum creatinine and albuminuria.^19^ The RI obtained in the intrarenal arteries is an indirect predictor of RAS with high sensitivity and specificity.^20^ The results of our study are consistent with a similar previous study that reported a significantly higher RI and renal volume in T2DM patients than in healthy individuals and confirmed the presence detectable haemodynamic changes on US imaging even in patients with normal or high GFR and no albuminuria.^21^ Bruno et al. reported that the dynamic US evaluation of the renal RI is an early detector of vascular alterations in T2DM even before the occurrence of microalbuminuria.^22^ Furthermore, Delsart et al. found that an elevated renal RI of more than 0.7 is an independent predictor of a first renal or cardiovascular event in T2DM patients.^23^

In this study, we found no significant difference in the RL between controlled and uncontrolled DM patients. This was explained by Umanath and Lewis, who reported that kidney size and weight increase by an average 15% in DM patients and remain increased even after a decrease in kidney function.^15^ In contrast, the renal RI differed significantly between controlled and uncontrolled DM patients. This is consistent with Chirinos and Townsend, who found that a high RI is associated with high HbA1c levels and a low GFR.^24^ HbA1c is a reliable measure of chronic glycaemia. It correlates with the risk of long-term complications and is considered the test of choice for monitoring DM control.^25^,^26^ This elucidates and highlights the importance of controlling DM to improve renal perfusion and subsequently reduce renal function complications.

A previous study reported that creatinine is a useful biochemical measure in approximating the GFR.^27^ In our study, serum creatinine was slightly higher in patients with uncontrolled DM than in those with controlled DM. This is indicative of the importance of controlling DM for the preservation of a normal GFR and renal function.

### Limitations of the Study

A limitation of this study is that creatinine levels were not measured in the control group. Therefore, creatinine comparisons between the DM and control groups were not performed. Another limitation is that renal artery diameter measurements could not be performed in all patients and controls due to technically difficulties in measuring it. Therefore, such comparisons were not performed. Further studies with comparing creatinine and diameter of renal arteries in the DM and control groups are recommended.

## CONCLUSION

T2DM has a significant accentuating effects on RL, RI and PI of the kidneys even before diagnosing AKI or CKD, that may be attenuated by careful regulation of DM which can be slowing disease progression and preventing renal failure. Ultrasound Doppler is a highly valuable imaging modality for evaluating the subtle effects of T2DM on kidney dimensions and blood flow. RI can be implemented as an important tool in early diagnosis of kidney disease.

### Authors’ contribution:

**AAA:** Provided US Doppler and collected data.

**SAA:** Organized and analyzed data and wrote the final draft of the article and is responsible for the integrity of the work.

**AAQ:** Revised the manuscript.

**AMH:** Interpreted data and revised the manuscript.

## References

[1] Rodriguez-de-Velasquez A, Yoder IC, Velasquez PA, Papanicolaou N (1995). Imaging the effects of diabetes on the genitourinary system. Radiographics.

[2] Bateman RM, Sharpe MD, Jagger JE, Ellis CG, Solé-Violán J, López-Rodríguez M (2016). 36th International Symposium on Intensive Care and Emergency Medicine:Brussels, Belgium. 15-18 March 2016 Crit Care.

[3] O'Neill WC (2000). Sonographic evaluation of renal failure. Am J Kidney Dis.

[4] Ahmed S, Bughio S, Hassan M, Lal S, Ali M (2019). Role of Ultrasound in the Diagnosis of Chronic Kidney Disease and its Correlation with Serum Creatinine Level. Cureus.

[5] Ablett MJ, Coulthard A, Lee RE, Richardson DL, Bellas T, Owen JP, Keir MJ, Butler TJ (1995). How reliable are ultrasound measurements of renal length in adults?. Br J Radiol.

[6] Braconnier P, Piskunowicz M, Vakilzadeh N, Müller ME, Zürcher E, Burnier M (2020). How reliable is renal ultrasound to measure renal length and volume in patients with chronic kidney disease compared with magnetic resonance imaging?. Acta Radiol.

[7] Viazzi F, Leoncini G, Derchi LE, Pontremoli R (2014). Ultrasound Doppler renal resistive index:a useful tool for the management of the hypertensive patient. J Hypertens.

[8] Petersen LJ, Petersen JR, Talleruphuus U, Ladefoged SD, Mehlsen J, Jensen HA (1997). The pulsatility index and the resistive index in renal arteries. Associations with long-term progression in chronic renal failure. Nephrol Dial Transplant.

[9] McArthur C, Geddes CC, Baxter GM (2011). Early measurement of pulsatility and resistive indexes:correlation with long-term renal transplant function. Radiology.

[10] Achamrah N, Colange G, Delay J, Rimbert A, Folope V, Petit A (2018). Comparison of body composition assessment by DXA and BIA according to the body mass index:A retrospective study on 3655 measures. PLoS One.

[11] Alicic RZ, Rooney MT, Tuttle KR (2017). Diabetic Kidney Disease:Challenges, Progress, and Possibilities. Clin J Am Soc Nephrol.

[12] Friedman AN, Wang J, Wahed AS, Docherty N, Fennern E, Pomp A (2019). The Association Between Kidney Disease and Diabetes Remission in Bariatric Surgery Patients With Type 2 Diabetes. Am J Kidney Dis.

[13] D'Agati VD, Chagnac A, de Vries AP, Levi M, Porrini E, Herman-Edelstein M (2016). Obesity-related glomerulopathy:clinical and pathologic characteristics and pathogenesis. Nat Rev Nephrol.

[14] Min TZ, Stephens MW, Kumar P, Chudleigh RA (2012). Renal complications of diabetes. British Medical Bulletin.

[15] Umanath K, Lewis JB (2018). Update on Diabetic Nephropathy:Core Curriculum 2018. Am J Kidney Dis.

[16] Zerbini G, Bonfanti R, Meschi F, Bognetti E, Paesano PL, Gianolli L (2006). Persistent renal hypertrophy and faster decline of glomerular filtration rate precede the development of microalbuminuria in type 1 diabetes. Diabetes.

[17] Toth-Manikowski S, Atta MG (2015). Diabetic Kidney Disease:Pathophysiology and Therapeutic Targets. J Diabetes Res.

[18] Afsar B, Elsurer R (2017). Increased renal resistive index in type 2 diabetes:Clinical relevance, mechanisms and future directions. Diabetes Metab Syndr.

[19] Shirin M, Sharif MM, Gurung A, Datta A (2015). Resistive Index of Intrarenal Artery in Evaluation of Diabetic Nephropathy. Bangladesh Med Res Counc Bull.

[20] Krumme B, Hollenbeck M (2007). Doppler sonography in renal artery stenosis--does the Resistive Index predict the success of intervention?. Nephrol Dial Transplant.

[21] Mancini M, Masulli M, Liuzzi R, Mainenti PP, Ragucci M, Maurea S (2013). Renal duplex sonographic evaluation of type 2 diabetic patients. J Ultrasound Med.

[22] Bruno RM, Daghini E, Landini L, Versari D, Salvati A, Santini E (2011). Dynamic evaluation of renal resistive index in normoalbuminuric patients with newly diagnosed hypertension or type 2 diabetes. Diabetologia.

[23] Delsart P, Vambergue A, Ninni S, Machuron F, Lelievre B, Ledieu G (2020). Prognostic significance of the renal resistive index in the primary prevention of type II diabetes. J Clin Hypertens (Greenwich).

[24] Chirinos JA, Townsend RR (2014). Systemic arterial hemodynamics and the “renal resistive index”:what is in a name?. J Clin Hypertens (Greenwich).

[25] Gardiner FW, Nwose EU, Bwititi PT, Crockett J, Wang L (2017). Does a hospital diabetes inpatient service reduce blood glucose and HbA1c levels?A prospective cohort study. Ann Med Surg (Lond).

[26] Sherwani SI, Khan HA, Ekhzaimy A, Masood A, Sakharkar MK (2016). Significance of HbA1c Test in Diagnosis and Prognosis of Diabetic Patients. Biomark Insights.

[27] Traynor J, Mactier R, Geddes CC, Fox JG (2006). How to measure renal function in clinical practice. BMJ.

